# Does Supplementary Information Add Value to Functional Food? Evidence from a Choice Experiment in China

**DOI:** 10.3390/nu14204424

**Published:** 2022-10-21

**Authors:** Yixing Tian, Hong Zhu, Honghua Chen

**Affiliations:** 1 College of Economics and Management, China Agricultural University, #17 Qinghua East Road, Haidian District, Beijing 100083, China; 2 Institute of Food and Nutrition Development, Ministry of Agriculture and Rural Affairs, #12 Zhong Guan Cun Southern Street, Haidian District, Beijing 100081, China

**Keywords:** information treatment, functional food, choice experiment, food attributes, willingness to pay

## Abstract

Establishing an effective choice architecture system enables people to improve their ability to make better food choices and encourage transformation of the food system into one that is more efficient, healthy, and sustainable. However, affecting consumer preferences by improving information supply is still a crucial issue that has not been comprehensively explored in China and many developing countries. This study aimed to identify the most effective information treatment method for increasing the likelihood of purchase and willingness to pay (WTP) for nutritionally enhanced eggs. A survey with five information treatments and a choice experiment was completed by a random sample of 2379 Chinese consumers, and the mixed logit model was subsequently applied to interpret the results. It was found that when nutritional information (NI), health benefit information (HBI), and/or market status quo information (MSQ) was presented to consumers, their utility increased. Different schemes had different effects on participants’ WTP. The HBI from scientific research institution, provided in the form of leaflets, has the most significant effect on improving WTP, increasing the WTP of consumers by 31.65%. WTP for functional eggs increased similarly in response to NI and MSQ information. However, adding NI to HBI did not significantly increase the value of functional eggs, especially when the information was presented to the interviewees in the form of short videos. This research broadens the present knowledge and application of an information communication strategy by suggesting that the combination of information content, carriers, source influence consumer preference and WTP for nutritionally enhanced eggs. The results have implications for the communication practices of food enterprises to optimize their marketing strategies and improve product innovation to add more value to the functional food.

## 1. Introduction

Improving a healthy diet has become an essential objective of public policy and has fostered the constant innovation of the food industry, and presents a challenge for countries with emerging economies and rapid urbanization in particular. According to a scientific research report on the Chinese Dietary Guidelines (2021), an unhealthy diet is the most crucial factor in the occurrence and death of chronic disease in China, and the unhealthy diet is responsible for the deaths of 3.1 million Chinese residents in 2017 [1]. In 2016, the Chinese Government issued the “Health China 2030” blueprint, which advocates the prevention of chronic diseases and encourages residents to adopt healthy eating and healthy habits [2].

The purpose of food is not only to fulfill hunger but to provide the human body with essential nutrients to prevent nutrition-related diseases and even improve physical and mental health [3,4]. Functional foods (nutritionally fortified foods) have been proved to play an outstanding role in this regard, and eggs are of particular interest from a functionality standpoint [5]. The Ministry of Agriculture and Rural Areas of China issued two national industry standards for nutritionally fortified eggs in 2022: (1) Omega-3 Polyunsaturated Fatty Acids Fortified Egg (NY/T 4069-2021); and (2) Technical Specification for Production of Omega-3 Polyunsaturated Fatty Acids Fortified Egg (NY/T 4070-2021) [6]. Although considerable efforts have been devoted to promoting the supply side, whether functional eggs can be commercially successful or not hinges on consumers’ cognition, perception, and reaction to this niche food product. Manufacturers and retailers have identified consumer preferences and willingness to pay (WTP) as crucial determinants of product adoption and promotion [7].

Previous studies have shown that sensory attributes, production methods, and nutritional properties (e.g., Omega-3-enriched) are the factors that most influence consumer preference for eggs [5,8,9]. In terms of nutrient properties, however, present research results are inconsistent. Palmieri et al. [10] surveyed 312 Italian consumers online and found a positive WTP and potential market development for functional eggs. Cao et al. [11] and Lusk [8], found that consumers in Canada and the U.S. were willing to pay a premium price for omega-3-enriched eggs. Żakowska-Biemans and Tekień [12] performed an investigation in Poland to verify if residents would accept foods combined with sustainability and nutrition claims. They found that people were willing to pay a premium price for eggs containing omega-3 fatty acids, as claimed. Yeh et al. [13] concluded that consumers in Hungary and Italy were prepared to pay more for eggs with nutritional fortification claims or labels. However, Ahmad Hanis et al. [9], and Heng et al. [14], found that consumers in the U.S. and Malaysia were reluctant to pay a certain premium for omega-3-enriched eggs. It was found that respondents may not be familiar with functional eggs, meanwhile, they may have limited knowledge of their benefits [5].

In the case of new food nutrient enhancement technology that the public may be unacquainted with, studies suggest that the type, source, and framing of information provided to the consumers will always influence the public perception of functional foods [15]. Studies have shown that providing nutritional information is regarded as a very beneficial measure by government agencies around the globe [16,17,18,19]. However, the nutritional information provided on back-of-pack nutrition facts panel and front-of-pack nutrition claims do not frequently assist consumers in making the best decision, since messages are not always easily understandable for most of the audience, especially in functional food [20]. Panzone, et al. [21] found that Supplementary Information, not the health claim, increased the WTP of consumers for nutritionally enriched eggs.

Chinese consumers largely disregard food labels due to a lack of subjective and objective knowledge of nutritional elements and health benefits [15]. Therefore, it is imperative to launch an information intervention to promote consumer understanding and confidence in utilizing nutritional labels and claims. To date, the impacts of information treatments on acceptance of, and preference for, functional eggs have not been extensively discussed in either developing or developed countries; and how consumers are simulated to purchase healthier food is far from being fully understood in China [15,16]. Overall, the content of existing information intervention at present is relatively simple. The intervention is mainly in the form of information cards [11,22,23,24], and rarely applies videos. In terms of intervention content, almost all studies focus on nutrition-related claims [21,25,26]. Few studies have attempted to consider whether consumers will change their purchase intention by referring to other people’s behavior of purchasing nutritionally fortified eggs.

Can information treatments supplement the insufficient cognition of Chinese consumers and increase the probability that consumers will purchase functional eggs? What informational content is most effective at altering consumer preferences? Through what channels can information intervention more effectively influence consumer decision-making? These essential questions have not been answered and adequately demonstrated. Thaler and Sunstein [27] found that a better selection system can assist people in developing their trade-off ability and making favorable choices, one of which is to make information about diverse alternatives more understandable. Based on this, this paper expands the nutritional composition table and the claims on the nutritionally fortified eggs currently sold in the current market into five different information intervention schemes, aiming to transform simple digital information into content that consumers can understand, adopt and implement more easily. Given the research gaps in consumer preferences for nutritionally fortified eggs and responses to information treatment, this research attempts to evaluate the influence of various information sources, content (nutritional content, health outcomes and market status quo), and information carriers (leaflets and videos) on consumer choice probability and WTP for nutritional enhancement through a rigorous economic experiment.

The launch of new niche foods developed by enhancing nutritious or fortifying functional ingredients to carrier foods on the market not only improves dietary nutrition and health of people but also provides producers with new business opportunities. This study makes several contributions to the existing literature and professional practice. Previous studies on the effect of an information intervention on consumer preferences began with a single treatment content and/or method and lacked a systematic analysis of intervention effects. The multi-angle treatment designs bring this study closer to the actual situation of consumer purchasing decisions, ensuring the effectiveness of the questionnaire and the accuracy of the results. In addition, mastering the influence of information treatment on consumer preferences will aid the government in formulating standards and related policies and provide a reference value for enterprises in formulating their marketing management. Nutritionally enhanced eggs are in a period of vigorous development. Measuring consumer preferences at this stage can effectively reflect the real demand side in the market, and understanding the impact of information treatment on consumer WTP is conducive to relevant enterprises in formulating marketing and publicity strategies.

## 2. Study Design

### 2.1. Survey Instrument

Data for this study were collected through a nationwide online survey administered by Wen Juan Xing Inc. (Shanghai, China), which is the largest professional questionnaire platform in China. The questionnaire was revised based on input from two rounds of pilot surveys. A total of 2500 participants from across the country were solicited randomly, and we finally received 2379 valid responses.

We employed two strategies in the survey to improve the reliability and quality of data. Following Lin and Nayga [28] and Elias, et al. [29], participants were screened out of the survey if they failed to promise to provide truthful responses at the beginning of the questionnaire. The method of trap questions, which were composed of two questions at 1/4 and 3/4 of the way through the questionnaire, was used to determine whether or not the participants were paying attention, respectively. We asked the participants to select the corresponding text name from three options based on the photographs of animals. Participants that failed the trap questions were removed from the sample. See Supplementary Materials Table S1 for the framework of questionnaire.

### 2.2. Information Treatments

Respondents were assigned randomly to either the informed groups (treatment groups) or the uninformed group (control group). In this survey, participants in the control group were not given any additional information before the choice experiment. This experiment included five intervention schemes. The content, source, and carrier of intervention information are distinct for each scheme. Table 1 summarizes the five treatment groups (1 in Section 7).

The information came from online social media and the scientific research institution (Institute of Food and Nutrition Development, Ministry of Agriculture and Rural Areas). In general, the information comprises scientific knowledge regarding: (1) the nutritional content information (NI) (2 in Section 7); (2) health benefits information of nutritionally enhanced elements (HBI) (3 in Section 7); (3) market status quo: the consumption of functional eggs by domestic and foreign consumers, and the national production standards of various countries (MSQ). Information treatment was distributed randomly to respondents through leaflets or recorded videos.

To examine the effect of information on consumer behavior, we must control the amount of information in each intervention group with reasonable precision. The crucial views expressed in leaflets and short videos regarding the same intervention content are identical. The only difference is that the language used in the video is more conversational and less academic. In addition to words, the leaflet contains charts. The time for respondents to receive information treatment in different schemes is controlled to be the same. Participants were unable to bypass the information intervention part within the specified two minutes, but they were permitted to re-read the leaflet or re-watch the video again at any time before answering the choice experiment questions.

The present study includes HBI and NI, respectively, as the intervention content in Treatment A (TA), Treatment B (TB), and Treatment C (TC). According to Ford et al. [30], consumers perceived health and nutritional claims independently, i.e., information about health benefits does not influence the processing of nutritional information. In this case, these two pieces of information each have a different and independent impact on consumer perceptions and decisions. These results are also confirmed by Jurkenbeck et al. [31], Barons et al. [32], and Franco-Arellano et al. [33]. Therefore, consumers in group A saw a leaflet with information from scientific research institutions about the health benefits of nutritionally fortified eggs. Participants in group B viewed a two-and-a-half-minute short video from the scientific research institution, which introduced both nutritional content and health outcomes of functional eggs (HBI and NI). The consumers of group C received a leaflet (the length of the text message is similar to that of group A) that detailed the functional ingredients and nutritional content (NI) of fortified eggs sold on the Chinese market and the difference between them and ordinary eggs.

According to Thaler and Sunstein [27], most people learn what they need from others, which is generally a good practice; thus, social influence is one of the most effective ways to nudge. The three social influences that Thaler and Sunstein [27] have highlighted—information, peer pressure, and priming—are usually utilized by both private and public nudgers. Based on this theory, we developed Treatment D (TD) and Treatment E (TE) to inform consumers of the current application of nutritional fortification technology in the United States, Europe, and Japan, the production standards and prices of nutritionally enhanced eggs, and household consumption in both developed countries and China (4 in Section 7), to satisfy consumer curiosity and interest in the consumption behaviors of other people in their daily lives. In addition, we also provide brands and channels for Chinese consumers to purchase nutritious eggs in the current market. Although TD and TE have different information sources and carriers, their content is similar.

### 2.3. Choice Experiment Design

A choice experiment (CE) was applied to examine consumer preferences, choice possibilities, and WTP for various attributes of functional eggs. A comprehensive review of egg market trends and existing literature on consumer preferences in China proceeded with the selection of attributes for this study [6]. The present survey includes five attributes: nutrition enrichment; organic certification; rearing conditions; brand; and price (Table 2).

Given the focus of this study, the claim of nutritional enhancement is a prerequisite. Among all nutritionally fortified eggs, omega-3-enriched eggs, selenium-enriched eggs, and folic acid-enriched eggs are the most popular in China. Previous research on the preference and WTP of Chinese consumers for eggs did not emphasize nutritional enhancement as an attribute sufficiently. This gap is filled by our study, which examines public demand for the three most commonly seen and purchased functional eggs in China.

The decision to have organic certification and rearing conditions as attributes of food safety, sustainability, and animal welfare is becoming a big concern and determinant of food quality [34]. It is believed that organic eggs have fewer chemical residues and have the potential to improve the environmental sustainability of agriculture. Most Chinese consumers believe free-range eggs are tastier than caged eggs as the hens have access to abundant natural feeds when they can run freely outside cages.

We regard brand familiarity as another group of attributes. It has been pointed out that consumers are loyal to brands they frequently purchase, and thus the brands are regarded as a search attribute that has a similar effect as certification labels or claims [35]. The present study divided the attributes of egg brands into “habitually purchased brands” and “non-habitually purchased brands” as Chinese consumers lack a stable and long-term functional egg purchase experience.

The role of price as an attribute is to generate a part-worth utility for calculating the WTP participants assigned to the presence of the other attributes. We also believe that when consumers face a relatively new product, the price can be a quality signal to help them make the purchase decision. The present survey set the price attribute ranging from CNY 1 per egg to CNY 4 per egg. The lowest and highest prices are obtained through field research before the present survey.

**Figure 1:**
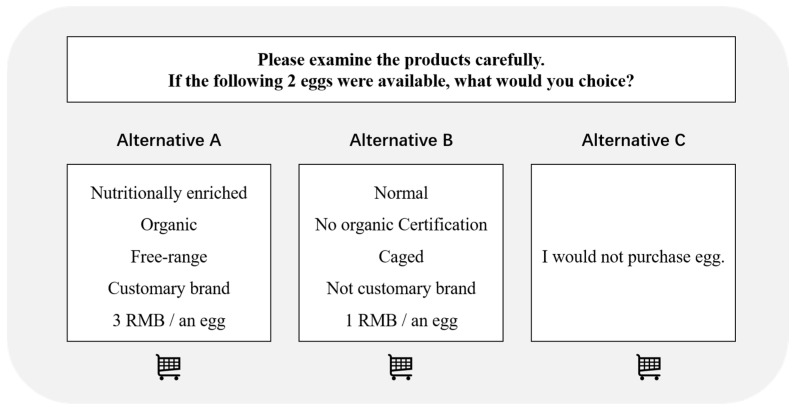
Example of a choice set in the survey.

The attributes and level settings lead to a total of (2 × 2 × 2 × 2 × 4)^2^ = 4096 possible choice sets in total. Our experiment uses a fractional factorial design that satisfies balanced and orthogonal. It is infeasible and overwhelming for a respondent to repeat too many alternatives within the limited time frame of the survey. The efficiency design was chosen based on the D-efficiency criterion [28]. Utilizing the %Mktblock Macro in the SAS software [36], we generated two eight-question blocks. In this case, each respondent only needs to complete eight choice tasks, randomly selected from one of two blocks. The D-efficiency score, which measures the quality and effectiveness of a design, was 100 (out of 100) for the final survey design. Each set has two product profiles (alternatives) with experimentally designed attribute levels and one null-purchase (opt-out) option. Adding a null-purchase alternative to the CE questions has been proved to improve the realism of the experiment [13]. A sample choice scenario is displayed in Figure 1.

To mitigate potential hypothetical bias, we provided participants with a cheap-talk script following Yeh, Menozzi and Török [13], and Lin and Nayga [28], before the CE questions. See Figure S1 in Supplementary Materials Figure S1 for the translation of the script. The questionnaire also includes a description of each attribute, which assists participants in understanding and evaluating the alternatives.

## 3. Theoretical Model and Econometric Specification

### 3.1. Conceptual Framework

**Figure 2:**
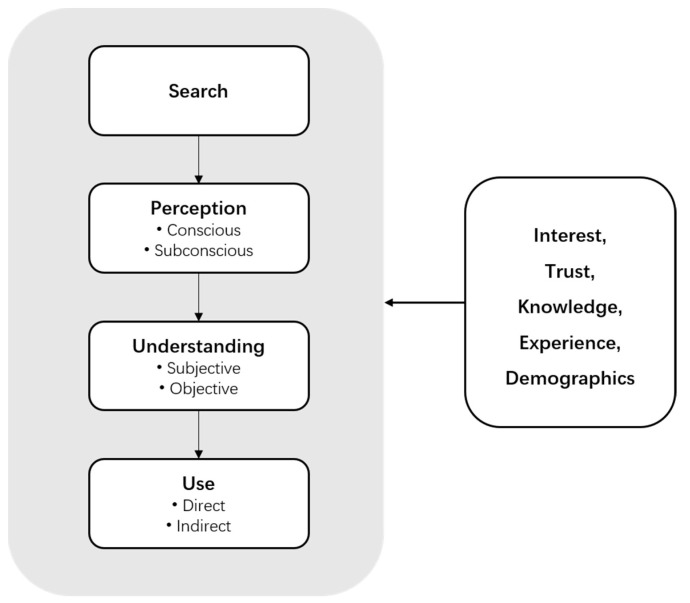
Consumer information processing model (adapted from Grunert and Wills (2007) [37]).

Figure 2 presents the information processing model adapted from Grunert and Wills [37] for this research. According to Thaler and Sunstein [27], there are two systems at work when people think and make decisions: the automatic system and the reflective system. Conscious perception is more like a reflective system, which may have stronger effects on the subsequent behavior [38]. Perception then leads to understanding, which includes both subjective and objective understanding.

Experience, interest, familiarity, and knowledge about products enable people to reduce confusion regarding general health and nutrition claims and play a vital role when consumers evaluate the credibility of any claims [38]. The knowledge of health-promoting foods has been proposed to heighten consumer interest in trying more innovative and nutritional foods according to Melo et al. [39]. In previous studies, subjective cognition is whether consumers understand the content of information, and objective cognition is whether their perception matches the scientific profile of health or nutritional claims [38].

In the present survey, all participants were required to respond to several questions, including a self-assessed subjective test of their knowledge of nutritionally fortified eggs on a scale ranging from “no knowledge (1)” to “very knowledgeable (5)”, and an objective test of their knowledge of fatty acids, selenium, folic acid consisting of five multiple-choice questions and five true or false questions. The knowledge assessment questions were developed based on previous studies by Altmann, et al. [40], Żakowska-Biemans and Tekień [12], and Peschel et al. [41], Liu, Hoefkens and Verbeke [42].

The random utility theory (RUT) for analyzing the choice experiment data was applied in this paper. Let $U_{ijn}$ represent consumer i’s utility by choosing the j-th product in the n-th choice question. Therefore, the utility function of product attributes $X_{ijn}$ can be expressed as

(1) $$U_{ijn}=\alpha X_{ijn}+\varepsilon_{ijn}$$

where a is a vector of unknown part-worth utility that is associated with attributes and $\varepsilon_{ijn}$ is a disturbance term satisfying independently identically distributed (i.i.d.).

### 3.2. Empirical Specification and Estimation

The mixed logit (ML) model is applied to the choice experiment data for analysis. In this study, we assume heterogeneity in preferences was irrelevant to observed characteristics [43]. The ML model has been proved to reflect a more realistic substitution pattern than standard logit models [44].

Under the utility maximization condition, a respondent chooses an alternative j in the n-th question only if it gives him/her the highest utility level compared to other alternatives offered in the question. Therefore, the choice probability is:

(2) $$Prob_{ij}=\int \frac{\exp\left(X_{ijn}\right)}{\sum_{j=1}^{4}X_{ijn}}f\left(\alpha \right)d\alpha$$

The associated log likelihood is given by:

(3) $$L=\sum_{i=1}^{I}\sum_{j=1}^{J}d_{ij}lnProb_{ij}$$

where, $d_{ij}=1$ if consumer i selects alternative j and zero otherwise; $Prob_{ij}$ is defined in Equation (2). The simulated maximum likelihood estimation method [44] with 1000 Halton draws was applied to estimate the parameters in Equations (2) and (3). Using the ML model, we analyse the data in the preference space to determine the choice probability and preference of respondents. The coefficients of the non-price attributes are assumed to be random and follow normal distributions since there is no evidence to confirm whether consumers have a positive or negative preference for these attributes. This model set is consistent with existing studies by Altmann et al. [40], and Britwum and Yiannaka [7].

The mixed logit model was applied in the WTP space to explore consumer WTP for various egg attributes and how information treatments affect the WTP of respondents vary with their understanding of different additional information by split samples. The model specified in the WTP space has significant advantages over the methods used by Altmann et al. [45], Markosyan, McCluskey and Wahl [23], as it avoids the arbitrary choice of the WTP distribution that results from dividing the coefficients of non-price attributes by the coefficient of price [43]. WTP space estimation allows price heterogeneity and calculates a more realistic WTP value [46].

Specifically, rewriting Equation (1) as a function of price and all other attributes, we could obtain:

(4) $$U_{ijn}=\alpha_{in}^{price}price_{in}+\alpha X_{ijn}+\varepsilon_{ijn}$$

where, $\alpha_{in}^{price}$ and α are individual-specific parameters for the price and the other non-price attributes, respectively. This results in a mixed logit model specified in preference space, in which the WTP is given for all non-price attributes [47], further rewriting Equation (4) as

(5) $$U_{ijn}=\alpha_{in}^{price}\left(price_{in}+\beta X_{ijn}\right)+\varepsilon_{ijn}$$

to yield a model, which is specified in WTP space. In this case, Equation (5) could be estimated based on simulated maximum likelihood methods to obtain consumer WTP for egg attributes in Stata.

Similar to other studies assessing the role of additional information in consumer preferences and choices [23,28,38,40], we apply a most preferred between-subjects experiment in behavioural economics to examine the effectiveness of different external information provision. Instead of within-subject experiments, between-subjects experiments would more accurately simulate a situation in which some consumers may be aware of a nutritional or health claim on the package or promotional video while others may not.

## 4. Survey Results

### 4.1. Sample Characteristics

The final samples were all consumers over 18 years old who participated in food consumption decision-making in their families. There were 2379 people in the pooled sample, including 1712 people (71.96%) who received information intervention. The remaining 667 people (28.04%) who received no additional information, were classified as part of the control group. The demographic characteristics are presented in Table 3.

### 4.2. Purchase Intention

Respondents were asked to evaluate their willingness to purchase three kinds of nutritionally fortified eggs. The question utilized a 5-point Likert scale anchored from “very reluctant” to “very glad”. Before the information intervention, respondents had an average score of 3.80 for willingness to purchase fortified eggs, close to “more likely” to purchase. After the information intervention, the average score increased by 3.16%, to 3.92. The vast majority of respondents were willing to give functional eggs a try.

**Figure 3:**
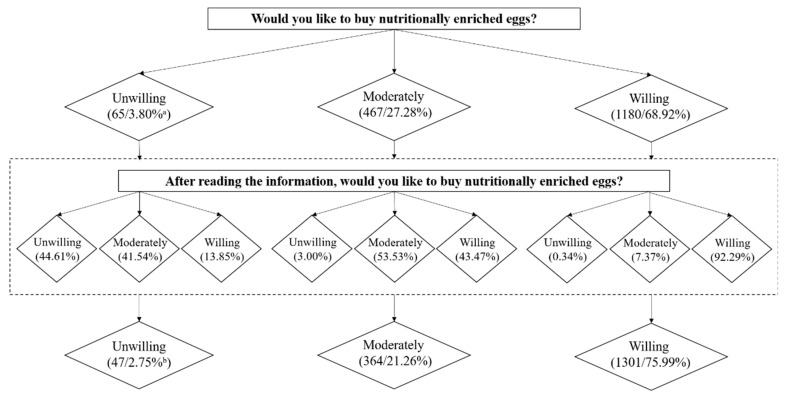
Respondents’ purchase intention before and after receiving the information. Note: ^a^, ^b^ the proportion was calculated according to the total number of people in the treatment groups (*n* = 1712). For example, 3.80% of participants, who were in treatment groups, were unwilling to try to purchase the nutritionally enriched eggs.

We combined the first two levels as “unwilling”, the third level as “moderately”, and the fourth and fifth levels as “willing” for statistical convenience. Figure 3 shows the structural change in the intentions of participants to purchase nutritionally fortified eggs. In general, 20.10% of the respondents in treatment groups changed their intention to purchase nutrition fortified eggs after the information intervention. Among them, 5.32%, 12.68%, and 2.10% of respondents were “willing to buy”, “moderately”, and “unwilling to buy”, respectively. This result shows that the role of information may differ for respondents with varying initial purchase likelihoods. The number of people whose initial intention is “unwilling” is significantly lower than that of other groups. Hence, those respondents whose initial intention was “unwilling” are more inclined to maintain their original attitude. Consumers with relatively neutral attitudes were more likely to be affected by information treatment.

### 4.3. Purchasing and Consumption Experience

The results show that compared to organic or free-range eggs, consumers lack purchase experience. A total of 86.09% of respondents had purchased free-range eggs, 42.71% had purchased organic eggs, and 34.51% of respondents had consumed at least one nutritionally enhanced egg. In the present survey, 139 respondents stated that they had consumed all three types of functional eggs, whereas 559 respondents indicated that they had consumed only one of the three nutritionally fortified eggs. Only nearly 4% of functional-egg experienced consumers regularly purchased in the past six months. The number of people who have purchased selenium-enriched eggs is greater than that of the other two types of nutritionally fortified eggs.

When asked why they were unwilling to purchase nutritionally fortified eggs, several respondents said they did not understand the nutritional content, efficacy, and value (71.56%). The number of people who decide to distrust the ability to supplement nutrients (59.56%) and safety (49.78%) ranks second and third, respectively. Some respondents indicated that their lack of understanding and concern regarding nutritional fortification technology (44.00%) diminished their willingness to purchase nutritionally fortified eggs. Few respondents indicated that fortified eggs were expensive (39.56%) and the purchase channels were inconvenient (18.22%).

### 4.4. Knowledge Score

The average self-assess subjective knowledge score is 2.69. Consumers have a general perception that they lack sufficient knowledge. Only 2.48% of participants think they fully understand and are well informed about functional eggs, while 10.59% of participants believe that they have no acquaintance with any knowledge about nutritionally fortified eggs. It was also found that the self-assessment of respondents with experience in purchasing nutritionally fortified eggs differ significantly from those of participants with no such experience. Experienced consumers believe they know more about nutritionally fortified eggs than those without any experience.

**Figure 4:**
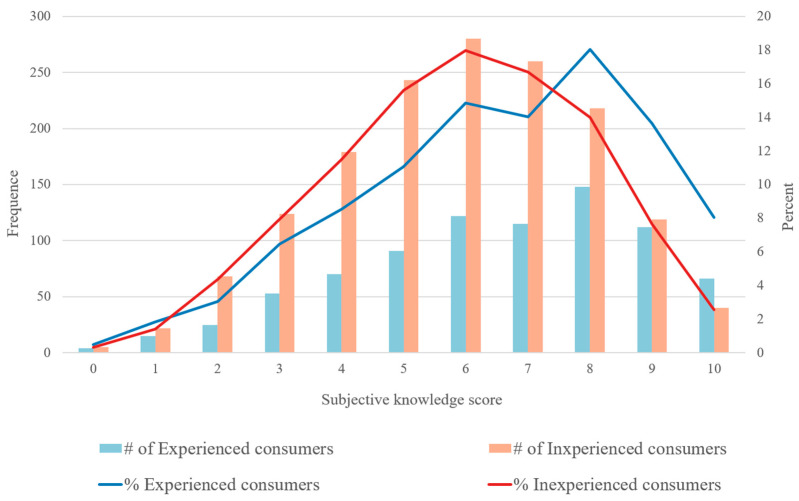
Distribution of subjective knowledge score based on consumption experience of functional eggs.

The mean objective knowledge score of experienced and inexperienced consumers are 6.51 and 5.89, respectively. The objective knowledge score of consumers with experience purchasing eggs with nutritional fortification is significantly higher than that of consumers without experience. Figure 4 shows the number and percent of experienced and inexperienced consumers among the different scores. The objective knowledge scores of consumers without purchase experience exhibit an approximate normal distribution. The scores of consumers with purchasing experience are relatively concentrated in segments with higher scores. More than 18% of experienced consumers scored eight points on the test, while nearly 18% of inexperienced consumers scored six points on the test.

## 5. Empirical Analysis and Discussion

### 5.1. Consumer Preference Estimations

Table 4 reports estimation results for the split sample of informed and uninformed consumers using the mixed logit model (Equation (2)) in preference space. Estimation of the model yields four crucial findings.

First, both in treatment groups (model 1–5) and control groups (model 6), most of the estimated attribute’s coefficients are highly significant and have the expected signs. More specifically, according to economic theory, the price signs for all groups are significantly negative, implying that consumer utility decreases with increasing price units. The coefficient of null is negative and statistically significant in both treatment and control groups, suggesting that respondents would derive a higher utility level from selecting any alternative other than “no purchase”.

Second, making health and/or nutrition claims or providing market status quo information would promote consumer valuation for nutritional enhancement. Aside from TD, the estimated coefficients of nutrition enhancement in both treatment and control groups are positive and statistically significant. This result implies that consumers would benefit more from choosing nutritionally fortified eggs over regular eggs. Moreover, the nutrition enhancement has a significant positive coefficient in the control group, suggesting that participants maintained a positive preference for nutrition enhanced eggs despite the absence of an additional information treatment. However, the magnitude of the coefficients is different between informed and uninformed groups. All enhancement coefficients in the treatment groups are greater than those in the control group, demonstrating that participants who received additional information have a higher utility level for nutritional fortification and are therefore more likely to select nutritionally enhanced eggs. This finding confirms the role of health and/or nutritional information in the consumer preference for nutritionally fortified eggs found previously by Żakowska-Biemans and Tekień [12], Ballco, Jurado and Gracia [38], Yeh, Menozzi and Török [13].

Third, the coefficients of organic certification and brand are all significantly positive. Additionally, all coefficients of free-range breeding are positive, and most subgroups are statistically significant. Meanwhile, organic eggs were generally preferred to free range eggs, although both production methods have their benefits. This result contradicts the findings of Żakowska-Biemans and Tekień [12], who found that free range eggs had a greater relative importance ranking than organic certification. According to the present survey and previous study [48], food safety remains the leading concern in China and is more likely to influence egg purchases than animal welfare. As a result, consumers are more interested in organic eggs than the free-range production system. The significant positive effect of habitual purchase brands indicates that respondents are more likely to purchase egg brands that they are familiar with and purchase frequently in daily life. This finding is consistent with previous research in China [6,35] and many other countries [45,49] that trust in one brand could be accumulated over time through regular purchases.

Finally, there is significant heterogeneity in consumer preferences for nutrition enrichment among Treatment A, B, and C. All of the standard deviation parameters for the attribute “nutrition enrichment”, regarded as random parameters, were significant in treatment groups A to C, indicating that consumers have a substantial heterogeneous preference regarding nutritional enrichment under respective treatments when purchasing eggs. In addition, the results reveal that there is no substantial heterogeneity in the preference for organic certification among the various groups.

### 5.2. Simulated Willingness-to-Pay

In this section, the WTP estimates of all informed and uninformed groups are compared to provide additional insights regarding the monetary value that consumers assign to their preference for nutritional enhancement. Table 5 reveals the results of estimating Equation (5) in WTP space for each treatment. The simulated WTP demonstrates the following crucial points.

First, respondents who received Supplementary Information are willing to pay 1.82 CNY for nutritionally enhanced eggs, 30.94% higher than that of the control group. It was found that providing respondents with information regarding HBIs, NIs, or MSQ increases their WTP by an average of 0.43 CNY. This result suggests that information treatment can effectively enhance the WTP of consumers. However, not all of the five information treatment plans can increase the WTP significantly compared with the control group.

From the perspective of the information carrier, the information treatment provided in the form of information leaflets has a more significant impact on improving WTP than the information treatment provided in the form of a short video. Consumers in Treatment A, C, and D are willing to pay CNY 1.83 and CNY 1.49, respectively, to buy nutritionally fortified eggs, which is greater than the WTP of the control group. Meanwhile, the intervention presented as a short video has no significant impact on increasing consumer WTP. For example, the respondents in Treatment B and E are willing to pay 1.21 and 1.37 CNY, respectively, for nutritional enhancement, which is less than the respondents in the control group. Several consumers (76.12%) believe that the information card posted on the product packaging or shelf is the better offline publicity. There is little difference in the number of people who select to place posters in the product sales area and play promotional videos on tablets.

Among the existing studies on the impact of a short video on the purchase intention of agricultural products in China, some studies have shown that it is more advantageous to stimulate the purchase of hedonic products (e.g., milk tea and chocolate) than functional products (e.g., bread, milk, and eggs) through short videos on social media [50,51]. People generally believed that, compared to the static display of commodity images, short videos are more stereoscopic and more effective at evoking the virtual touch of consumers through their visual and auditory perceptions. However, the usability of information from a short video damages the virtual haptics (5 in Section 7) of people according to the research by Guo, et al. [52].

In our experiment, short videos containing health and nutrition claims and market information were presented as “speech” or “interviews”. The short videos used in the experiment did not include any production process of functional eggs, nor a more engaging live video, particularly in Treatment B. Hence, Treatments B and E that utilized short videos as information carriers lacked sufficient interactivity to affect consumer emotional experience. In addition, we found that respondents needed to view an entire two-minute video to understand all the information regarding nutrition and health benefits and the current market status quo. However, the respondents in Treatment A, C, and D could instantly extract the information they were interested in through the keywords by glancing at the information leaflet. Compared to leaflets, short videos require more time and consistent attention from respondents to obtain the missing information in their cognition in a short time. Therefore, contrary to conventional cognition, short videos do not improve the generation of consumer virtual touch to stimulate and enhance consumer WTP.

Third, the impact of different types of information contained on consumer WTP is distinct. Consumers are willing to pay a premium to be informed about the health benefits of enhanced ingredients (HBI), rather than the amount of nutrient enrichment in products (NI). One explanation is that respondents employ the availability heuristic proposed by Thaler and Sunstein [27] in decision-making in the context of the choice experiment after the information treatment. In Treatment A, we provided respondents with numerous real-world examples to explain why selenium and folic acid supplementation is necessary. For example, “Chinese residents mainly eat cooked vegetables. Too much oil, too fine knife skill, too fierce heat all lead to the loss of folic acid in food”. In addition, we provide channels for the absorption of unsaturated fatty acids in daily life, such as “to supplement omega-3 fatty acids, you can eat fish directly, or choose flaxseed, flaxseed oil, perilla, and other foods rich in α- Linolenic acid”. Respondents were able to easily connect with their own lived experiences and evaluate possible health risks and potential health benefits on relevant examples provided in the treatment materials.

Meanwhile, the significant increase in WTP for functional eggs in Treatment D relative to the control group demonstrates that information about MSQ exerts a significant impact on nudging consumers to embrace nutritional enhancement. Consumers who have received information about the consumption of nutritionally fortified eggs by residents of other countries, the price of eggs, and the government established production standards are also willing to pay a certain amount of extra money for the nutritional enhancement. The influence of society or the market on consumer dietary behavior can take many forms: one person can be influenced by what others consume, and cooking culture can spread from one culture to another [53]. The results of this study confirm the findings of previous research, that the norms formed by the actual choices of others influence our behavior [6].

Fourth, an intriguing finding is that simultaneously providing nutritional content and health benefits information to consumers does not significantly improve their WTP. Treatment B has a lower WTP value than Treatments A and C separately. When confronted with more complex information to be processed, participants with a cognitive burden may be more likely to stick with the status quo or their pre-existing cognition [54]. Ballco, Jurado and Gracia [38] found that health claims did not add value to the associated nutritional claim regarding fibre enrichment in foods, whereas the health claim regarding the reduction in saturated fat added value to the associated nutritional claim. The essence of this conflicting conclusion is that “avoiding harm” is more crucial than “seeking benefits”. It is worth noting that the research of Ballco, Jurado and Gracia [38] is only based on and limited to the application of nutritional and health claims. In our experiment, the intervention materials did not completely differentiate between “eliminating a particular risk” and “obtaining a particular function”. When we replace the research object with information treatments that are richer in content and more complex in structure, the plausibility of this explanation remains debatable. Actually, many studies have shown that consumers are not more rational when given more information about food purchases [28,37,55]. Thaler and Sunstein [27] found that an automatic system, as mentioned in Section 3.1, can be exercised through repeated reading and processing of information. Therefore, compared to reading two kinds of intervention information simultaneously, it is more effective to constantly repeat one statement (health benefit or nutrition content) through various examples and explanations to increase consumer awareness and link information in their minds to better understand our treatment and make choices quickly.

Fifth, in terms of information sources, the treatment improves consumer WTP more effectively when the information comes from experts or scientific research institutions, although this effect is limited. The results show that the information offered by experts from the Institute of Food and Nutrition Development can effectively improve consumer WTP (${\mathrm{WTP}}_{\mathrm{Treatment}\;A}>{\mathrm{WTP}}_{\mathrm{Control}}$), but the WTPs of respondents in Treatments B and E are lower than anticipated. On the contrary, the information from social media and manufacturers has improved the WTP of consumers to a certain extent, relative to the uninformed participants (${\mathrm{WTP}}_{\mathrm{Treatment}\;C}>{\mathrm{WTP}}_{\mathrm{Treatment}\;D}>{\mathrm{WTP}}_{\mathrm{Control}}>{\mathrm{WTP}}_{\mathrm{Treatment}\;E}>{\mathrm{WTP}}_{\mathrm{Treatment}\;B}$).

## 6. Conclusions

Acceptance and preference for food nutrition fortification technology by consumers have become crucial determinants for the successful adoption of new technologies by producers and retailers. This study investigated the effect of five different information treatment plans elicitation on consumer WTP for nutritional enhancement attributes as both behavioral policy tools and marketing means that influence individual decision-making.

The present survey highlights that experienced participants are more knowledgeable about the nutrition quality and health benefits of unsaturated fatty acids, folic acid, and selenium than consumers who have never made a purchase. Consumers with relatively neutral attitudes were more likely to be affected by information treatment, demonstrating that information intervention may be more effective and successful with consumers who have not formed stable preferences and habits.

The estimation results of mixed logit models indicate that information treatment contributes to promote consumer preferences for functional eggs. The information, including nutrition content and/or health benefit information or market status quo, has a positive impact on consumer choice probability of purchasing nutritionally fortified eggs regardless of their form or carrier. The result of this study informs policy makers that the use of particular nutrition education among the general public through information treatment could be effective in encouraging the choice of novel and healthy food products. From the perspective of agribusiness and supply-chain management, providing consumers with sufficient information about health efficacy, nutritional content, and marketing can increase consumer WTP.

Among the three information contents, the publicity of health benefits may have a more apparent effect on improving WTP for nutritional enhancement than the other two. Since the health benefits information does not add value to the nutritional content information, the amount of information that functional food companies provide to their consumers is another important issue that affects the purchase intention. Functional food enterprises can frequently popularize the knowledge of the relationship between nutritional elements and human health through articles, blogs, brochures, and other channels. However, the length of information printed on food packaging needs to be strictly controlled. Notable is the fact that the results of this study highlight the importance of social impact. Consumers are easy to follow the buying behavior of people around them. Therefore, providing information regarding the purchase of nutritionally fortified foods and product prices by other consumers will improve the WTP of target customers.

In addition, the carrier of intervention information also needs to be carefully selected and allocated. In contrast to the current popular short video platform publicity represented by TikTok, this experiment found that the information intervention in the form of a short video had no significant effect on consumer real-time food purchase decisions. Based on this conclusion, providing a certain amount of information on the leaflet is more effective for immediately influencing consumer purchase decisions. Short video content must be more novel and engaging if sellers want to promote or publicize functional products used daily. In fact, the rapid rise and development of the live broadcasting industry in the context of the Internet has provided an updated channel for information intervention. Sellers may be willing to show feed production or chicken raising through live broadcasting, which will have a more direct and vivid impact on consumers’ cognition and preferences.

This study result shows that although food scientists are believed to be credible, they are not always the best communicators. Food scientists can provide professional knowledge, but professional communicators must also be involved. Developing an easy and operable intervention may lead us to focus too much on the content of information that needs to be conveyed while ignoring who and how to convey it. In the future, a more effective and comprehensive information treatment will require the collaboration of the government, scientific research institutions, enterprises, and social groups in multiple directions and multiple topics to scientifically guide consumers to select foods with a higher nutritional intensity.

A note on the limitations of this study is in order before closing. Consistent with several studies examining consumer preferences, we were unable to convince participants to spend money on functional eggs during the survey, preventing us from observing valuable feedback on participants’ valuation on more eggs’ attributes such as flavor and color of egg yolk. Due to space limitations, consumer attitudes towards both functional foods and information treatment are not included in the present analysis. However, we have started the investigation of consumer heterogeneity, looking forward to providing more powerful support for the design of information treatment in future papers.

## 7. Notes

1. Detailed context of the information provided under each treatment group are available on request from the corresponding author due to the limited length requirements of the paper.
2. The content includes the following aspects: (1) the specific nutrient content of eggs enriched in unsaturated fatty acids, selenium, and folic acid, respectively (e.g., the content of DHA, EPA, and ALA per 100 g of omega-3-enriched eggs); (2) the comparison of omega-3, selenium, and folic acid contents between functional eggs of a brand and daily foods or ingredients (such as lamb, dried abalone, pork); (3) the appropriate intake (AI) of nutrient elements introduced or suggested by the Food and Agriculture Organization of the United Nations (FAO, Rome, Italy), the European Food Safety Administration (EFSA, Parma, Italy), and the Chinese Nutrition Society (CNS, Beijing, China), respectively.
3. The HBIs provide additional information by stating: (1) the health benefits of the enrichments; (2) manifestations of nutritional deficiency and possible adverse effects on the body; (3) specific indications of factors (e.g., lifestyle and eating habits) associated with a deficiency in unsaturated fatty acids, selenium, and folic acid. For example, omega-3 unsaturated fatty acids have physiological functions such as regulating blood lipids, clearing thrombus, enhancing immunity, maintaining the retina, notifying the brain, and improving joint inflammation. In a healthy diet, the appropriate proportion of omega-6 and omega-3 is 4~6:1. Selenium deficiency is a significant factor in the development of Kashan disease and Kashan–Beck disease. Folic acid deficiency can lead to megaloblastic anemia, fetal malformation, and neural tube development defects.
4. The data on Chinese consumer purchase frequency mainly comes from our survey on the consumption of nutritionally fortified eggs in Beijing, Shanghai, Guangzhou, and Xi’an in 2020.
5. Virtual haptics refers to a tactile experience that closely resembles a real-world scene, which people form based on the tactile memory information they have accumulated in the past.

## Figures and Tables

**Table 1:** Description of treatment groups.

Scheme	Intervention Content	Information Source	Information Carrier
TA	HBI	Scientific research institution	Leaflet
TB	HBI + NI		Short video
TC	NI	Web	Leaflet
TD	MSQ	Web	Leaflet
TE		Scientific research institution	Short video
Control	/	/	/

Note: TA, TB, TC, TD, TE refer to Treatments A, B, C, D, E, respectively. HBI refers to health benefit information, NI refers to nutritional information, MSQ refers to market status quo. Treatments A and C communicate only the health benefits and nutritional content information, respectively, via leaflets, while Treatment B communicates both the nutritional content and the associated health benefit via short video. Treatments D and E transmit information about market status quo both in China and around the world via leaflet and video, respectively. The respondents were informed that the information of Treatments A, B and E comes from scientific research institutions, while Treatments C and D come from the web.

**Table 2:** Attributes and levels used in the choice experiment design.

Attributes	Levels	Description
Nutrition Enrichment	Enriched	Refers to whether the egg is enriched with omega-3, selenium, or folic acid.
	Normal *	
Organic Certification	Organic	Refers to whether the egg has an organic certification on the package.
	Conventional *	
Rearing Conditions	Free-range	Refers to whether the egg is caged-free or not.
	Caged *	
Brand	Habitual purchase brands	Refers to whether it is a brand that consumers are familiar with and often buy.
	Not habitual purchase brands *	
Price	1 CNY	Refers to price for per egg in the market where the respondents typically shop.
	2 CNY	
	3 CNY	
	4 CNY	

Note: * represents the base level.

**Table 3:** Social demographic and economic characteristics, by experimental condition.

Variables	Pool	TA	TB	TC	TD	TE	Control
Sample size	2379	335	333	333	332	379	667
Female [8.779 (0.118)] ^a^	1383	189	207	195	197	198	397
Age [28.274 (0.103)]							
18–34	1807	240	249	259	256	283	520
35–44	400	67	61	57	44	67	104
45–54	128	20	18	14	20	25	31
55–64	36	8	3	2	8	4	11
>65	8	0	2	1	4	0	1
Education [15.612 (0.111)]							
High school	142	17	15	20	20	25	45
Bachelor’s degree	1879	259	271	262	255	318	514
Graduate degree or above	358	59	47	51	57	36	108
Income level [52.311 (0.211)]							
<50 thousands CNY	127	23	15	20	14	19	36
50–100 thousand CNY	324	36	55	45	39	58	91
110–150 thousand CNY	421	47	50	63	65	73	123
160–200 thousand CNY	461	72	61	52	58	77	141
210–300 thousand CNY	506	80	66	78	70	81	131
310–400 thousand CNY	246	39	41	32	41	32	61
410–500 thousand CNY	145	18	22	18	23	22	42
510–700 thousand CNY	83	6	12	17	13	12	23
710 thousand–1 million CNY	41	5	10	4	6	4	12
>1 million CNY	25	9	1	4	3	1	7
Marital status [27.876 (0.002)]							
Single	858	121	130	121	123	96	267
Married without children	250	40	31	37	36	39	67
Married with children	1271	174	172	175	173	244	333

Note: ^a^ Chi-square test statistics (*p*-value) testing the null hypothesis of indifference between groups of variables across treatments.

**Table 4:** Estimation results of the mixed logit model, by treatment.

Variables	(1)	(2)	(3)	(4)	(5)	(6)
	TA	TB	TC	TD	TE	Control
Mean values						
price	−0.528 ***	−1.299 **	−0.591 ***	−0.673 ***	−0.654 ***	−0.452 ***
	(0.169)	(0.589)	(0.132)	(0.241)	(0.245)	(0.090)
enhanced	1.233 **	1.463 ***	1.125 ***	1.097	0.984 **	0.724 ***
	(0.570)	(0.552)	(0.296)	(0.748)	(0.453)	(0.160)
organic	0.756 **	2.173 **	0.735 ***	0.944 **	0.957 **	0.857 ***
	(0.313)	(0.882)	(0.197)	(0.462)	(0.452)	(0.158)
cage free	0.555	1.130 **	0.799 ***	0.802	0.694 **	0.449 ***
	(0.379)	(0.478)	(0.266)	(0.551)	(0.353)	(0.140)
brand	1.381 **	2.078 **	1.366 ***	1.418 **	1.279 **	1.151 ***
	(0.645)	(0.850)	(0.318)	(0.693)	(0.550)	(0.221)
null	−28.989 ***	−37.945 ***	−29.111 ***	−33.865 ***	−29.396 ***	−27.554 ***
	(4.744)	(9.824)	(2.561)	(4.898)	(2.555)	(0.945)
Standard deviation of parameter distribution						
enhanced	1.506 ***	3.562 **	1.322 **	−0.109	0.004	−0.601
	(0.414)	(1.673)	(0.614)	(0.455)	(0.770)	(0.710)
organic	−0.673	−0.317	−0.938	−0.995	−1.293	−0.482
	(2.559)	(0.305)	(0.723)	(2.193)	(1.534)	(0.437)
cage free	3.266 *	3.433 **	−3.024 ***	3.231	2.787 ***	2.012 ***
	(1.723)	(1.681)	(1.059)	(2.255)	(0.954)	(0.557)
brand	0.257	−4.216 **	0.583	1.852 **	1.215	1.300 ***
	(0.241)	(2.111)	(0.367)	(0.926)	(1.229)	(0.438)
Log likelihood	−1227.377	−1235.939	−1182.999	−1214.002	−1387.958	−2513.742
Observations	6879	6822	6600	6852	7794	13,638

Notes: Standard errors in parentheses. * *p* < 0.1, ** *p* < 0.05, *** *p* < 0.01.

**Table 5:** Simulated WTP (CNY per egg) for different attributes across treatments and control group.

Variables	(1)	(2)	(3)	(4)	(5)	(6)
	TA	TB	TC	TD	TE	Control
enhanced	1.83 ***	1.21 ***	1.49 ***	1.49 ***	1.37 ***	1.39 ***
	(0.210)	(0.182)	(0.194)	(0.182)	(0.172)	(0.118)
organic	1.54 ***	1.50 ***	1.52 ***	1.52 ***	1.52 ***	1.57 ***
	(0.073)	(0.071)	(0.072)	(0.072)	(0.072)	(0.076)
cage free	0.51 ***	0.45 ***	0.48 ***	0.48 ***	0.48 ***	0.60 ***
	(0.091)	(0.087)	(0.090)	(0.090)	(0.089)	(0.097)
brand	1.92 ***	1.83 ***	1.87 ***	1.86 ***	1.86 ***	1.99 ***
	(0.138)	(0.131)	(0.134)	(0.133)	(0.132)	(0.139)
Log likelihood	−9044.4541	−9096.1459	−9077.3501	−9081.3046	−9071.6455	−9062.1337

Notes: Standard errors in parentheses. *** *p* < 0.01. The actual WTP of TC and TD is 1.494 and 1.486 for enhanced. However, since the secondary currency unit of RMB is two digits after the decimal point, the figures of WTP in this table are obtained by rounding the measured value to two decimal places.

## Data Availability

The data presented in this study are available on request from the corresponding author. The data are not publicly available due to the reports using the same set of data for analysis has not yet been published.

## Supplementary Materials

The following supporting information can be downloaded at: https://www.mdpi.com/article/10.3390/nu14204424/s1, Figure S1: Cheap talk script; Table S1: Framework of the questionnaire.

## References

[1] Chinese Nutrition Society (2021). The Chinese Dietary Guidelines.

[2] Healthy China Action Promotion Committee "Health China 2030" Blueprint. http://www.gov.cn/xinwen/2019-07/15/content_5409694.htm.

[3] Siró I., Kápolna E., Kápolna B., Lugasi A. (2008). Functional food. Product development, marketing and consumer acceptance—A review. Appetite.

[4] Annunziata A., Vecchio R. (2013). Consumer perception of functional foods: A conjoint analysis with probiotics. Food Qual. Prefer..

[5] Rondoni A., Asioli D., Millan E. (2020). Consumer Behaviour, Perceptions, and Preferences towards Eggs: A review of the Literature and Discussion of Industry Implications. Trends Food Sci. Technol..

[6] Tian Y., Zhu H., Zhang L., Chen H. (2022). Consumer preference for nutritionally fortified eggs and impact of health benefit information. Foods.

[7] Britwum K., Yiannaka A. (2019). Consumer willingness to pay for food safety interventions: The role of message framing and issue involvement. Food Policy.

[8] Lusk J.L. (2019). Consumer preferences for cage-free eggs and impacts of retailer pledges. Agribusiness.

[9] Hanis I.A.H.A., Nasir S.M., Jinap S., Alias R., Ab Karim M.S. (2013). Consumer’s preferences for eggs attributes in Malaysia: Evidence from conjoint survey. Int. Food Res. J..

[10] Palmieri N., Stefanoni W., Latterini F., Pari L. (2022). Factors Influencing Italian Consumers’ Willingness to Pay for Eggs Enriched with Omega-3-Fatty Acids. Foods.

[11] Cao Y., Cranfield J., Chen C., Widowski T. (2021). Heterogeneous informational and attitudinal impacts on consumer preferences for eggs from welfare enhanced cage systems. Food Policy.

[12] Żakowska-Biemans S., Tekień A. (2017). Free Range, Organic? Polish Consumers Preferences Regarding Information on Farming System and Nutritional Enhancement of Eggs: A Discrete Choice Based Experiment. Sustainability.

[13] Yeh C.-H., Menozzi D., Török Á. (2020). Eliciting Egg Consumer Preferences for Organic Labels and Omega 3 Claims in Italy and Hungary. Foods.

[14] Heng Y., Peterson H.H., Li X. (2013). Consumer Attitudes toward Farm-Animal Welfare: The Case of Laying Hens. J. Agric. Resour. Econ..

[15] Huang L., Bai L., Gong S. (2020). The effects of carrier, benefit, and perceived trust in information channel on functional food purchase intention among Chinese consumers. Food Qual. Prefer..

[16] Guan L., Zhang Y., Jin S., Zhou L. (2021). Understanding the low use rate of food nutrition information in China. Int. Food Agribus. Manag. Rev..

[17] Delivett C.P., Farrow C.V., Thomas J.M., Nash R.A. (2022). Front-of-pack health imagery on both ‘healthy’ and ‘unhealthy’ foods leads people to misremember seeing health claims: Two memory experiments. Appetite.

[18] Chevallier H., Herpin F., Kergosien H., Ventura G., Allaert F.A. (2021). A Graded Approach for Evaluating Health Claims about Plant-Based Food Supplements: Application of a Case Study Methodology. Nutrients.

[19] Mazzu M.F., Baccelloni A., Finistauri P. (2022). Uncovering the Effect of European Policy-Making Initiatives in Addressing Nutrition-Related Issues: A Systematic Literature Review and Bibliometric Analysis on Front-of-Pack Labels. Nutrients.

[20] Gonzalez-Diaz C., Vilaplana-Aparicio M.J., Iglesias-Garcia M. (2020). How Is Functional Food Advertising Understood? An Approximation in University Students. Nutrients.

[21] Panzone L., Garrod G., Adinolfi F., Pasquale J.D. (2022). Molecular marketing, personalised information and willingness-to-pay for functional foods: Vitamin D enriched eggs. J. Agric. Econ..

[22] Castellari E., Ricci E.C., Stranieri S., Marette S., Sarnataro M., Soregaroli C. (2019). Relationships between health and environmental information on the willingness to pay for functional foods: The case of a new aloe vera based product. Nutrients.

[23] Markosyan A., McCluskey J.J., Wahl T.I. (2009). Consumer Response to Information about a Functional Food Product: Apples Enriched with Antioxidants. Can. J. Agric. Econ. Rev. Can. Agroecon..

[24] Tian Y., Croog R., Bovay J., Concepcion A., Getchis T.L., Kelly M.R. (2022). Who responds to health, environmental, and economic information about local food? Evidence from Connecticut seafood consumers. Aquac. Econ. Manag..

[25] Naspetti S., Alberti F., Mozzon M., Zingaretti S., Zanoli R. (2020). Effect of information on consumer preferences and willingness-to-pay for sparkling mock wines. Br. Food J..

[26] Ahn B.-I., Bae M.-S., Nayga R.M. (2016). Information Effects on Consumers’ Preferences and Willingness to Pay for a Functional Food Product: The Case of Red Ginseng Concentrate. Asian Econ. J..

[27] Thaler R.H., Sunstein C.R. (2009). Nudge: Improving Decisions about Health, Wealth and Happiness.

[28] Lin W., Nayga R.M. (2022). Green identity labeling, environmental information, and pro-environmental food choices. Food Policy.

[29] Elias J.J., Lacetera N., Macis M. (2019). Paying for Kidneys? A Randomized Survey and Choice Experiment. Am. Econ. Rev..

[30] Ford G.T., Hastak M., Mitra A., Ringold D.J. (1996). Can consumers interpret nutrition information in the presence of a health claim? A laboratory investigation. J. Public Policy Mark..

[31] Jurkenbeck K., Mehlhose C., Zuhlsdorf A. (2022). The influence of the Nutri-Score on the perceived healthiness of foods labelled with a nutrition claim of sugar. PLoS ONE.

[32] Barons K.P., Mann D., Orellana L., Miller M., Pettigrew S., Sacks G. (2022). Nutrition-Related Information on Alcoholic Beverages in Victoria, Australia, 2021. Int. J. Environ. Res. Public Health.

[33] Franco-Arellano B., Bernstein J.T., Norsen S., Schermel A., L’Abbe M.R. (2017). Assessing nutrition and other claims on food labels: A repeated cross-sectional analysis of the Canadian food supply. BMC Nutr..

[34] Li R., Lee C.-H., Lin Y.-T., Liu C.-W. (2020). Chinese consumers’ willingness to pay for organic foods: A conceptual review. Int. Food Agribus. Manag. Rev..

[35] Wang E.P., Liu Z.Z., Gao Z.F., Wen Q., Geng X.H. (2022). Consumer preferences for agricultural product brands in an E-commerce environment. Agribusiness.

[36] SAS Institute Inc. (2013). SAS 9.4.

[37] Wills J.M., Bonsmann S.S.G., Kolka M., Grunert K.G. (2012). European consumers and health claims: Attitudes, understanding and purchasing behaviour. Proc. Nutr. Soc..

[38] Ballco P., Jurado F., Gracia A. (2020). Do Health Claims Add Value to Nutritional Claims? Evidence from a Close-to-real Experiment on Breakfast Biscuits. Food Qual. Prefer..

[39] Melo G., Zhen C., Colson G. (2019). Does point-of-sale nutrition information improve the nutritional quality of food choices?. Econ. Hum. Biol..

[40] Altmann B.A., Anders S., Risius A., Mörlein D. (2022). Information effects on consumer preferences for alternative animal feedstuffs. Food Policy.

[41] Peschel A.O., Grebitus C., Steiner B., Veeman M. (2016). How does consumer knowledge affect environmentally sustainable choices? Evidence from a cross-country latent class analysis of food labels. Appetite.

[42] Liu R., Hoefkens C., Verbeke W. (2015). Chinese consumers’ understanding and use of a food nutrition label and their determinants. Food Qual. Prefer..

[43] Hole A.R., Kolstad J.R. (2012). Mixed logit estimation of willingness to pay distributions: A comparison of models in preference and WTP space using data from a health-related choice experiment. Empir. Econ..

[44] Train K.E. (2009). Discrete Choice Methods with Simulation.

[45] Ahmad W., Anders S. (2012). The Value of Brand and Convenience Attributes in Highly Processed Food Products. Can. J. Agric. Econ..

[46] Balogh P., Bekesi D., Gorton M., Popp J., Lengyel P. (2016). Consumer willingness to pay for traditional food products. Food Policy.

[47] Li S., Zhu C., Chen Q., Liu Y. (2019). Consumer confidence and consumers’ preferences for infant formulas in China. J. Integr. Agric..

[48] Cao M. (2021). Research of Consumer Preference and Motivation for Animal Product from Different Housing Systems: Case of Egg Products. Master’s Thesis.

[49] Rather R.A., Hollebeek L.D., Tan V.T., Ramkissoon H., Leppiman A., Smith D. (2022). Shaping customer brand loyalty during the pandemic: The role of brand credibility, value congruence, experience, identification, and engagement. J. Consum. Behav..

[50] Wang Q. (2021). Research on the Impact of Content Characteristics of Short Video Advertisements on Consumers’ Impulsive Purchase Intention on Social Media. Master’s Thesis.

[51] Du R., Wang R. (2022). Analysis on the Influence of Short-form Video on Consumers’ Purchase Intention of Fresh Agricultural Products. Tianjin Agric. Sci..

[52] Guo H., Zhao Y., Shi H. (2019). Research of the Influence of Short-form Video Display on Customers’ Purchase Intention on the E-commerce Platform. Inf. Stud. Theory Appl..

[53] Roberto C.A., Kawachi I. (2015). Behavioral Economics and Public Health.

[54] Hensher D.A., Balbontin C., Collins A.T. (2018). Heterogeneity in decision processes: Embedding extremeness aversion, risk attitude and perceptual conditioning in multiple process rules choice making. Transp. Res. Part. A-Policy Pract..

[55] Saarela A.-M. (2014). Change of behaviour when selecting food products in a supermarket environment after reminding consumers about weight management. Public Health Nutr..

