# Supplementary material for: Does Supplementary Information Add Value to Functional Food? Evidence from a Choice Experiment in China
Source: Nutrients. 2022 Oct 21;14(20):4424. doi: 10.3390/nu14204424 (PMC9606922; doi:10.3390/nu14204424)
Supplement: Supplementary file 1 [file nutrients-14-04424-s001.zip › Supplementary-Table S1.pdf]

Table S1: Example questions of survey questionnaire

The first part of the questionnaire concerns the daily purchase experience of eggs and self-reported perceived importance of various aspects. The second part is the cognitive test of nutritional elements, nutritional fortification technology, dietary nutrition cognition. The third part is the information treatment experiment (the participants in the control group were not shown any intervention information). The fourth part is the choice experiment. The fifth part is the survey on demographic information.

Section 1: Daily Purchase

Q1: What types of eggs have you heard of? [Multiple Choice]

Q2: What types of eggs have you purchased (bought) in the last six months? [Multiple Choice]

Q3: What are the eggs you often buy in the last six months? [Single Choice]

Q4: What types of eggs have you consumed (eaten/taken) in the last six months? [Multiple Choice]

Options	Q1	Q2	Q3	Q4
A. Selenium enriched eggs<br>B. Omega-3 enriched eggs<br>C. Ordinary eggs<br>D. Organic eggs<br>E. Folic acid enriched eggs				

Q5: You've heard of functional eggs, but why didn't you buy them? [when Q1=A/B/E and Q2≠A/B/E]

A. I know nothing about the nutritional content, efficacy, and value.

B. I do not trust the functional eggs are able to supplement nutrients to human body.

C. I do not trust the safety of functional eggs.

D. I know nothing about the nutritionally fortification technology.

E. Functional eggs are expensive to me.

F. I do not know where to buy.

Q6: What factors do you consider when buying egg products? [Multiple Choice]

Q7: What is the most important consideration? [Single Choice]

Options	Q5	Q6
A. Date of manufacture<br>B. Expiration date<br>C. Brand<br>D. Place of Origin<br>E. Freshness<br>F. Package<br>G. Price<br>H. Promotion/Discount<br>I. Nutritive value		

J. Animal welfare in production process<br>K. Food safety certification<br>L. Egg appearance<br>M. Purchase channel<br>N. Taste<br>O. Government regulation<br>P. Others		

Q8: Are you willing to try to buy functional eggs (such as "selenium enriched eggs", "omega-3 eggs", "folic acid eggs", etc.) in the future? [Single Choice]

A. Very reluctant

B. Less likely

C. Moderately

D. More likely

E. Very glad to try

Trap Question#1

Section 2: Cognition Test

Q1: Do you know the function of unsaturated fatty acids? [Single Choice]

Q2: Do you know the function of folic acid? [Single Choice]

Q3: Do you know the function of selenium? [Single Choice]

Options (1 ~ 5 indicates the degree of what you know)	Q1	Q2	Q3
A. Not at all (1)<br>B. Do not quite well know (2)<br>C. Not sure (3)<br>D. A little bit (4)<br>E. Fully aware of (5)			

Q4: ____ deficiency is an important cause of Creutzfeldt-Jakob disease, macrosomia, and lower immunity.

A. Selenium

B. Zinc

C. Folic acid

D. Iron

Q5: Which of the following options is NOT a deficiency of folic acid?

A. Endemic goiter

B. Megaloblastic anemia

C. Cleft lip and palate

D. Neural tube malformation

Q6: Omega-3 unsaturated fatty acids DO NOT include which of the following options?

A. DHA

B. EPA

C. ALA

D. RDA

Q7: Do you think it is correct to say that "Omega-3 and Omega-6 are both unsaturated fatty acids, so either one can be supplemented"?

A. Correct

B. Wrong

C. Unsure

Q8: Do you think it is correct to say that "the principles of nutritional fortification and genetic modification are the same"?

A. Correct

B. Wrong

C. Unsure

Q9: Do you trust the nutrient contents have health benefits to human body? 1 means very distrustful and 5 means very trustful. How much do you trust? Please fill in the blank_____.

Q10: Are you aware of and do you agree with the following statements?

Statements	Agree	Disagree	Do not know
Fat and cholesterol are harmful to the body and should be avoided.			
Unsaturated fatty acids are more beneficial to human health than saturated fatty acids.			
Excessive pork intake can increase the risk of obesity, type 2 diabetes, and other morbidity.			
Nuts are rich in lipids and polyunsaturated fatty acids, protein, and are a useful addition to the diet.			
Deep-sea fish has a higher proportion of polyunsaturated fatty acids in the overall fat content compared to regular red meat, which encourages increased dietary intake of seaweed and fish.			

Section 3: Information treatment

Section 4: Choice experiment

Trap question #2 and Repeat Section 2

Section 5: Demographic information

Q1: What is your gender? [Single Choice]

A. Female
B. Male
C. Others

Q3: What is your age? ______

Q4: What is your education level (including ongoing education)? [Single Choice]
A. High school
B. Bachelor degree
C. Graduate degree or above

Q5. What is your total annual household income (including all bonus and other additional income)? [Single Choice]
A. Below ￥50,000
B. ￥50,000 - ￥100,000
C. ￥110,000 - ￥150,000
D. ￥160,000 - ￥200,000
E. ￥210,000 - ￥300,000
F. ￥310,000 - ￥400,000
G. ￥410,000 - ￥500,000
H. ￥510,000 - ￥700,000
I. ￥710,000 - One million
J. One million above

Q6: What's your family status? [Single Choice]
A. Single
B. Married without children
C. Married and have children

Q7: Are you usually involved in making decisions about your family's daily food consumption?
A. Yes
B. No

Note: *The above questions were translated from the actual research questionnaire. There are still some questions in the actual research questionnaire that involve data that have not yet been published and therefore are not included in this sample questionnaire.*
