# Supplementary material for: Does Supplementary Information Add Value to Functional Food? Evidence from a Choice Experiment in China
Source: Nutrients. 2022 Oct 21;14(20):4424. doi: 10.3390/nu14204424 (PMC9606922; doi:10.3390/nu14204424)
Supplement: Supplementary file 1 [file nutrients-14-04424-s001.zip › Supplementary-Figure S1.pdf]

Supplementary Material

Please read the information below carefully.

This part is a test of consumers' willingness to pay. No on will actually be paid money based on the decision you make, but you are asked to make the decision as though it would result in the actual payment. Such surveys tend to lead to the inconsistency between "willing to purchase" and "actual purchase". We call this a "hypothetical bias". In order to avoid this bias, please imagine that you are buying eggs in the supermarket when you answer the following questions.

"If I decide to choose option A (or option B), I must pay for it."

Figure S1 Cheap talk script
